# Cases of consecutive ductal adenocarcinoma of the prostate carrying HRR mutation: case series and literature review

**DOI:** 10.1093/omcr/omae124

**Published:** 2024-10-26

**Authors:** Weizhe Han, Nihati Rexiati, Yongzhi Wang, Tao Liu, Yongwen Luo, Zhonghua Yang

**Affiliations:** Department of Urology, Zhongnan Hospital of Wuhan University, 169 Donghu Road, Wuhan 430071, China; Department of Urology, Zhongnan Hospital of Wuhan University, 169 Donghu Road, Wuhan 430071, China; Department of Urology, Zhongnan Hospital of Wuhan University, 169 Donghu Road, Wuhan 430071, China; Department of Urology, Zhongnan Hospital of Wuhan University, 169 Donghu Road, Wuhan 430071, China; Department of Urology, Zhongnan Hospital of Wuhan University, 169 Donghu Road, Wuhan 430071, China; Department of Urology, Zhongnan Hospital of Wuhan University, 169 Donghu Road, Wuhan 430071, China

## Introduction

DAP is the second most often histologic subtype of prostate cancer (PCa). It is often mixed with acinar adenocarcinoma (AAP) and presented in 2.6% of PCa while pure DAP is even more rare, accounting for only 17 per 10 thousands of PCa [1].

It was reported that alterations in HRR-related genes are presented in about 23%–31% of patients with advanced PCa [2]. Patients with advanced disease carry mutations in the HRR pathway benefit from poly ADP-ribose polymerase inhibitors (PARPi) based on a so-called mechanism of synthetic lethality [3]. Here we present 6 consecutive cases of DAP recently diagnosed in our center, of which 5 patients were sequenced and all carried HRR-related gene alterations.

## Case presentation

### Case 1

A 59-year-old man complaining of dysuria with an history of HIV. The total and free serum prostate-specific antigen (PSA) were 3.899 ng/ml (0–4.0 ng/ml) and 1.411 ng/ml (0–0.934 ng/ml), respectively. Computed tomography urography (CTU) demonstrated a mass located at bladder neck, 10 × 9 × 10 mm in size, and magnetic resonance imaging (MRI) of the pelvis showed thickened urethra with nodules and significant enhancement throughout the whole urethra, suggesting the possibility of granulomatous lesions in the posterior urethra. Transurethral resection of bladder tumor (TURBT) detected DAP invading bladder and radical cystectomy (RC) was performed. Pathological examination revealed mixed prostate adenocarcinoma (Gleason score, GS 4 + 4), of which 80% of tumor was DAP and 20% of AAP. DNA sequencing identified CDK12 and BRCA1 mutations (Table 1). Androgen deprivation therapy (ADT) combined with PARP inhibitor were administrated 6 weeks postoperatively although the PSA was undetectable.

**Table 1:** Clinical characteristic and major mutations in HRR

**Case**	**Age (years)**	**1** ^**st**^ **line therapy**	**Response to 1** ^**st**^ **therapy**	**2** ^**nd**^ **line therapy**	**Respons to 2** ^**nd**^ **therapy**	**Type of mutation**	**Overall survival**
1	59	Radical cystectomy + ADT + PARPi	SD	No	Can not be assessed	BRCA1^exon12^-IGR^RPS6KC1^, IGR ^CCDC200^-BRCA1^exon13,^ CDK12^c.365dup(p.L122Ffs*5^, CDK12^c.2603_2609 + 98del^	Alive
2	75	Radical prostatectomy	Lung metastasis 5 months postoperately	ADT + AA	SD	CHEK2 ^c.973C > A (p.H325N)^	Alive
3	72	Radical prostatectomy + ADT + RT	Lymph node metastasis 15 months postoperately	ADT + AA	SD	ATM ^c.8572_8584 + 26del^	Alive
4	68	Radical cystectomy	Pelvic recurrence 15 months postoperately	ADT + AA	Lymph node metastasis 15 months on 2^nd^ therapy	BRCA1^M1628T^	65 months
5	62	Radical cystectomy + ADT + AA	SD	No	Can not be assessed	BRCA2 ^N2374S^	Alive
6	70	No	Lost to follow-up			DNA sequencing not performed	Lost to follow-up

ADT = androgen deprivation therapy; PARPi = poly ADP-ribose polymerase inhibitors; SD = stable diseas; AA = abiraterone acetate.

### Case 2

A 75-year-old man presented with dysuria and frequency with a PSA value of 2.78 ng/ml. TURP was performed for lower urinary tract symptoms (LUTS) and pathology examination revealed PDA (GS 4 + 4). RP was performed 4 weeks later and pathology result demonstrated features of mixed prostate carcinoma, of which DAP and AAP accounting for 85% and 15% of tumor, respectively. DNA sequencing identified a mutation of CHEK2. This patient experienced persistent PSA (0.17 ng/ml) and biochemical recurrence (BCR) 8 weeks and 5 months postoperatively, respectively. CT scan detected several lesions in both lungs, then ADT combination with abiraterone acetate (AA) were administrated because of low abundance of mutation.

### Case 3

A male patient of 72-year-old presented with frequency and urgency and has a history of hypertension. The total PSA was 9.69 ng/ml (0–4 ng/ml) with a f/t ratio of 0.13. MRI of the prostate demonstrated diffuse mixed signals and several node projecting into the bladder from the prostatic transitional zone. Prostate biopsy revealed prostate acinar adenocarcinoma (GS 4 + 4), however, the pathology was confirmed to be PDA (GS 4 + 5) after RP with a final stage of pT4N1M0. Subsequent DNA sequencing identified ATM mutation. The patient received salvage radiotherapy combination with ADT 6 weeks postoperatively for a PSA value of 2.25 ng/ml.

### Case 4

A 68-year-old man presented with gross hematuria with a PSA level of 11.068 ng/ml and CTU demonstrated a lesion in the bladder neck and trigone. Prostate biopsy and TURBT revealed prostate adenocarcinoma and subsequent radical cystoctomy demonstrated mixed prostate adenocarcinoma, most of which was DAP (GS 5 + 4). The patient received no further treatment 4 weeks postoperatively when PSA remained at 2.036 ng/ml and developed metastasis in both lungs one year later.

### Case 5

A man of 62-year presented with frequency and dysuria and an elevated value of PSA (191 ng/ml). Pelvic MRI demonstrated diffuse abnormal signals in prostate and seminal vesicles, and biopsy of prostate revealed DAP (GS 4 + 4). Routine radiography did not detect metastasis and RP was performed. Final pathologic examination demonstrated mixed prostate adenocarcinoma, DAP accounting for 90% of resected prostate. DNA sequencing identified a mutation of BRCA2.

### Case 6

A man of 70 years presented with gross hematuria. The total PSA was normal (8.415 ng/ml) and a f/t ratio of 0.73. CTU and pelvic MRI demonstrated lesions in the prostate and bladder neck. Although subsequent TURBT revealed mixed prostate adenocarcinoma (75% DAP) invading the muscle layer of the bladder, the simultaneous 13-core prostate biopsy could not detect any tumor.

## Discussion

DAP exhibits non-typical clinicopathological and radiological features, and compared with AAP, DAP is reported to have a significantly lower PSA levels at diagnosis [4], which make it difficult to be diagnosed at early stage [5]. In our series of 6 consecutive patients, two and four patients presented with PSA levels less than 4 ng/ml and 10 ng/ml, respectively, and 3 of 6 were diagnosed by TURBT or TURP for presenting with bladder lesions (Case 1, 6) or LUTS (Case 2). Only 1 case was directly diagnosed DAP by prostate biopsy (Case 5). As DAP often originates from primary periurethral prostate ducts, patients often present with LUTS or/and hematuria. In our series, 3 of 6 patients presented with dysuria and frequency, while 2 of 6 complaining of gross hematuria. Specially in case 6, we detected DAP by TURBT but got no signal of tumor by prostate biopsy. This can be partly explained that as described above that DAP often originates from primary periurethral prostate ducts surrounding urethral where is detoured in the procedure of prostate biopsy.

Liu Y et al. reported a 3%–3.8% patients of non-metastatic prostate cancer harbored somatic of germline aberrations in HRR genes [6]. Recently, Kobayashi H [7] and Seipel AH [8] reported the prevalence of HRR mutation in their reports of DAP as 0/11 and 1/11, respectively. To the best of our knowledge, it is the highest prevalence of HRR-related mutations reported in our series. As 5 of the 5 patients (5/5), all at non-metastatic stage, carried HRR-related mutations, including BRCAs in three patients, ATM, CHEK2 and CDK12 in one patient.

Compared with AAP of same clinical stage, DAP is usually associated with a worse prognosis for poor response to ADT and chemotherapy. These patients receiving conventional therapy, including RP or RT had worse outcomes than AAP patients although receiving neoadjuvant or adjuvant ADT [9, 10]. In our series, all cases experienced recurrence and metastasis, except for case 1 who had received adjuvant therapy of ADT combination with PARP inhibitor for more than 34 months. We postulate that the worse prognosis of DAP may partly be attributed to the higher prevalence of HRR mutation.

In conclusion, DAP should be diagnosed carefully even with a “normal” PSA level. Because DAP responses to ADT and chemotherapy poorly, personalized or precision therapies, including PARP inhibitor, may play an important role in this rare type of prostate cancer.
